# Plates for the Illustration of the Development, Both Natural and Abnormal of Man and Mammalia

**Published:** 1844-07

**Authors:** 


ART. III.—*Tabulæ ad illustrandam Embryogenesin Hominis et Mammalium, tam naturalem quam abnormem.* Auctore W. VROLIK, Med. Doctore, &c. Fasciculus I.—*Amstelodami*, 1844.

*Plates for the Illustration of the Development, both natural and abnormal, of Man and Mammalia.* By W. VROLIK, M.D., Professor in the Athenæum at Amsterdam, &c. Fasciculus I, with Five Plates, Folio. —*Amsterdam, London, Dublin, &c.* 1844.

IN a former Number we mentioned Dr. Vrolik's Dutch work, on the Natural and Morbid Development of the Fœtus. The first volume alone had then appeared, as the first of a large work on the whole subject of morbid anatomy. Since that time, the second volume of the same work, published in 1842, has appeared. In it, the morbid anatomy of congenital defects is completed, in a manner which fully justifies the high opinion we expressed of the merits of the first volume, and which places the work beyond comparison with any other on the same subject. Our readers will be glad to hear that a German translation of the work is in course of preparation, through which its contents will be accessible to many more than they could be in their original language.

The work now before us, by the same author, though illustrative of that just mentioned, will yet be complete in itself. It will consist of about a hundred plates, to be published quarterly, most of them being from original drawings of the preparations in the author's, and the other rich museums of Holland. The plates included in this, the first fasciculus, illustrate the formation of the decidua and corpus luteum, the fœtal circulation, complete ectopia of the thoracic and abdominal viscera, with a cloacal condition of the pelvic organs, and a congenital hernia through the diaphragm; the intention being, in each fasciculus, to publish some of the illustrations of both the natural and the anormal states. They are all accurate, and are lithographed with a remarkable clearness and delicacy of touch, three of the five being by Mayer, by whom the author's beautiful plates of the Chimpansi were drawn. The descriptions, in Latin as well as in Dutch, are distinct and sufficiently detailed. The work will be too much "a great work" for us to recommend it to all our readers; but all who are interested in the subject should possess it, and so should all who admire "great works" of whatever kind.

